# Complete Mitochondrial Genome of the Medicinal Mushroom *Ganoderma lucidum*

**DOI:** 10.1371/journal.pone.0072038

**Published:** 2013-08-26

**Authors:** Jianqin Li, Jianhui Zhang, Haimei Chen, Xiangdong Chen, Jin Lan, Chang Liu

**Affiliations:** Institute of Medicinal Plant Development, Chinese Academy of Medical Sciences, Beijing, P. R. China; University Paris South, France

## Abstract

*Ganoderma lucidum* is one of the well-known medicinal basidiomycetes worldwide. The mitochondrion, referred to as the second genome, is an organelle found in most eukaryotic cells and participates in critical cellular functions. Elucidating the structure and function of this genome is important to understand completely the genetic contents of *G. lucidum*. In this study, we assembled the mitochondrial genome of *G. lucidum* and analyzed the differential expressions of its encoded genes across three developmental stages. The mitochondrial genome is a typical circular DNA molecule of 60,630 bp with a GC content of 26.67%. Genome annotation identified genes that encode 15 conserved proteins, 27 tRNAs, small and large rRNAs, four homing endonucleases, and two hypothetical proteins. Except for genes encoding *trnW* and two hypothetical proteins, all genes were located on the positive strand. For the repeat structure analysis, eight forward, two inverted, and three tandem repeats were detected. A pair of fragments with a total length around 5.5 kb was found in both the nuclear and mitochondrial genomes, which suggests the possible transfer of DNA sequences between two genomes. RNA-Seq data for samples derived from three stages, namely, mycelia, primordia, and fruiting bodies, were mapped to the mitochondrial genome and qualified. The protein-coding genes were expressed higher in mycelia or primordial stages compared with those in the fruiting bodies. The rRNA abundances were significantly higher in all three stages. Two regions were transcribed but did not contain any identified protein or tRNA genes. Furthermore, three RNA-editing sites were detected. Genome synteny analysis showed that significant genome rearrangements occurred in the mitochondrial genomes. This study provides valuable information on the gene contents of the mitochondrial genome and their differential expressions at various developmental stages of *G. lucidum*. The results contribute to the understanding of the functions and evolution of fungal mitochondrial DNA.

## Introduction

*Ganoderma lucidum*, the symbol of traditional Chinese medicine, is one of the well-known medicinal basidiomycetes worldwide and is widely referred to as the “Mushroom of Immortality.” *G. lucidum* was first recorded in “Shen Nong Ben Cao Jing” [1], the earliest pharmacopoeia in China. The description of its pharmacological applications can be dated back to nearly two thousand years ago. *G. lucidum* is widely cultivated in Asian countries, including China, Japan, and South Korea. The annual sale of products derived from *G. lucidum* is estimated to be more than 2.5 billion U.S. dollars. The *Ganoderma* genus has approximately 51 species with similar morphological characteristics. The lack of powerful molecular makers for the determination of these species has caused wide mislabeling and misuse of *Ganoderma* products, which represent a threat to product safety [2]. We recently reported the complete genome sequence of *G. lucidum* (monokaryotic strain G.260125-1, which was derived from the dikaryotic strain CGMCC5.0026 by protoplasting) to elucidate the molecular mechanisms underlying the synthesis of diverse secondary metabolites in medicinal fungi and to lay the foundation for future studies on the evolutionary diversities of *Ganoderma* species [3]. However, the mitochondrial genome, also called the second genome, has not been studied in detail.

The mitochondrion is a membrane-enclosed organelle found in most eukaryotic cells [4]. Besides supplying cellular energy, mitochondria are involved in other tasks, such as signal translation, cellular differentiation, cell death, cell cycle, and cell growth control [5]. Elucidating the structure and function of the mitochondrion is important in understanding the genetic content of a fungus. A typical fungal mitochondrial genome contains 14 conserved protein-coding genes and a variable number of tRNA-coding genes [6], [7]. The mitochondria of filamentous fungi have uniparental inheritance and their genomes evolve faster than the corresponding nuclear DNA, thereby making them more suitable to differentiate closely related organisms [7], [8], [9]. The analysis of mitochondrial DNA (mtDNA) is useful in evolution and population studies at taxonomic levels below order [10], [11], [12], [13], [14], [15].

With the development of novel sequence methods, the number of mitochondrial genomes of fungi has increased. As of December 31, 2012, a total of 113 fungal mitochondrial genomes have been listed at the National Center for Biotechnology Information Organelle Genome Resources. Most of the species listed are represented by ascomycetes; basidiomycetes contain only 13 members. These genomes vary in size from 18,844 bp in *Hanseniaspora uvarum* to 127,206 bp in *Chaetomium thermophilum*. The mtDNA divergence between different fungal species is predominantly associated with variations in intergenic regions, intronic sequences, and gene orders, whereas the core protein-coding genes are conserved [15].

*G. lucidum* is a complex species; thus, distinguishing the sub-species is difficult. Understanding the relationship between cellular growth and development and the production of diverse bioactive compounds is highly interesting. Given the wide use of mitochondrial genome sequences in evolution and population studies and their biological function in providing energy needed for cellular growth, we characterized the genetic contents and differential gene expression of the mitochondrial genome of *G. lucidum*. The complete mitochondrial genome sequence of the *G. lucidum* strain CGMCC5.0026 was determined and analyzed to investigate its gene contents and repeats and to identify the open reading frames (ORFs) in the intergenic regions and intronic sequences. Transcriptomic (RNA-Seq) data were used for gene annotation, expression analysis, and detection of transcriptionally active regions (TARs). A comparative analysis of gene orders of this genome and other closely related species was performed to provide new insights on the evolution of basidiomycete mtDNA.

## Materials and Methods

### Assembly of Mitochondrial Genome of *G. lucidum*

Our previous paper described the complete genome sequence of *G. lucidum* by using the Roche 454 GS FLX (Roche, USA) and Illumina GA II (Illumina, USA) next generation sequencing (NGS) platforms [3]. We downloaded the mitochondrial genome of *Trametes cingulata* as a reference sequence for the mitochondrial genome assembly to identify the mitochondrial genome of *G. lucidum* [16]. *T. cingulata* is the closest relative of *G. lucidum* that has a complete mitochondrial genome available. Three large contigs obtained by whole genome de novo assembly were found similar to the genome sequence of *T. cingulata*. The sizes of these three contigs were 43,808, 7,650, and 7,509 bp. To fill the gap, these three contigs were used to search against 454 reads obtained from the total DNA. Reads similar to the ends of the three contigs were added stepwise by using the Seqman software (Lasergene 9.0, DNAStar, WI, USA). After multiple iterations, a single contig with a total length of 60,630 bp was obtained. This contig was inspected for complete coverage by 454 reads. Three regions with low coverage were further verified using polymerase chain reaction (PCR) amplification and DNA sequencing. Table S1 shows the primers used.

### Annotation of the Mitochondrial Genome

Annotation of the mitochondrial genome was initially conducted using M3T (http://www.herbalgenoics.org/m3t). ORFs were searched using the CLC sequence viewer version 6.5.1 (CLC bio, Aarhus, Denmark). The predicted gene models were manually edited using the published mitochondrial genome of *T. cingulata* as reference [16]. The module in the Sequence Manipulation Suite Version 2 (SMS2, http://www.bioinformatics.org/sms2/) was used to identify the hypothetical proteins. Proteins with length greater than 100 amino acids were retained for further analysis. Codon usage was determined using another module of SMS2. tRNA genes were initially identified using tRNAscan-SE [17] and verified by ARAGORN [18].

### Repeat Structure and Sequence Analysis

REPuter [19] was used to identify and locate the dispersed repeats, including the direct (forward) and inverted (palindrome) repeats. The repeat was not less than 90% identity (hamming distance equal to 3), with a size of more than 30 bp. Tandem repeats were analyzed using the Tandem Repeat Finder program [20] with advanced parameter settings 2, 7, and 7 for match, mismatch, and indels, respectively. The minimum alignment score and maximum period size were set at 50 and 500, respectively. After program analysis, the tandem repeats with less than 15 bp in length and the redundant results of REPuter were manually removed. An online system “Ori-Finder” was used to predict the origin of replication (oriC) [21].

### Transcriptome Sequencing and Analysis

RNA-Seq (deep-sequencing of cDNA) analyses for the transcriptomes of the three developmental stages of *G. lucidum* were previously performed and described [3]. The reads from the three different phases (mycelia, primordia, and fruiting bodies) were mapped to the mitochondrial genome assembly using Bowtie 2 [22], [23] with default settings. SeqMonk software (v0.22.0) (http://www.bioinformatics.babraham.ac.uk/) was used for the integration of genome features and gene expression, quantification of RNA abundance, and differential expression analysis. TARs were defined as any tract of >150 nucleotides (nt) with a minimum coverage of at least four reads per nt, at least 150 nt away from an annotated gene [24].

### Real-time Quantitative PCR (RT-qPCR)

Total RNAs were extracted from three different developmental stages (mycelia, primordia, and fruiting bodies) by using an RNeasy Plant Mini Kit (QIAGEN, USA). The total RNA samples were digested with DNase I and reverse transcribed into single-stranded complementary DNA (TaKaRa, Japan). RT-qPCR was performed thrice for each sample by using SYBR green (TaKaRa, Japan) on the ABI PRISM 7500 Real-Time PCR System (Life Technologies, USA). The expression data for the genes were normalized against that of *rns*. Table S1 shows the primers used.

### Phylogenetic Analysis Method

The amino acid sequences of the protein-encoding genes *atp6*, *atp8*, *atp9*, *cob*, *cox1*, and *cox2* were used for phylogenetic analysis. These sequences were present in all of the following 17 species: *G. lucidum*, *Moniliophthora roreri*, *Moniliophthora perniciosa*, *Lentinula edodes*, *Pleurotus ostreatus*, *Schizophyllum commune*, *T. cingulata*, *Cryptococcus neoformans*, *Phakopsora pachyrhizi*, *Phakopsora meibomiae*, *Ustilago maydis*, *Tilletia walkeri*, *Tilletia indica*, *Saccharomyces cerevisiae*, *Schizosaccharomyces japonica*, *Schizosaccharomyces pombe*, and *Schizosaccharomyces octosporus*. The sequences of the selected proteins in other fungi were extracted from the M3T web server. The evolutionary history was inferred using the Maximum Likelihood method based on the JTT matrix-based model. The bootstrap consensus tree inferred from 1000 replicates was obtained to represent the evolutionary history of the taxa analyzed. Evolutionary analysis was conducted using MEGA5 [25].

### Comparison of Global Genome Structure

The mitochondria-conserved sequences were identified between *G. lucidum* and *T. cingulata*, *S. commune*, *C. neoformans*, and *U. maydis* by BLASTN with an e-value cutoff of 1e-10. The synteny and annotation files were uploaded to a web-based genome synteny viewer GSV [26].

### Data Availability

The mtDNA sequence of *G. lucidum* strain CGMCC5.0026 was deposited in EMBL under the accession number HF570115. The RNA-Seq reads were deposited in the short-read archive at GenBank under the accession number SRA048015.The transcriptomic data also can be downloaded from our interactive web portal at http://www.herbalgenomics.org/galu [3].

## Results

### I. Genome Organization

**Figure 1:**
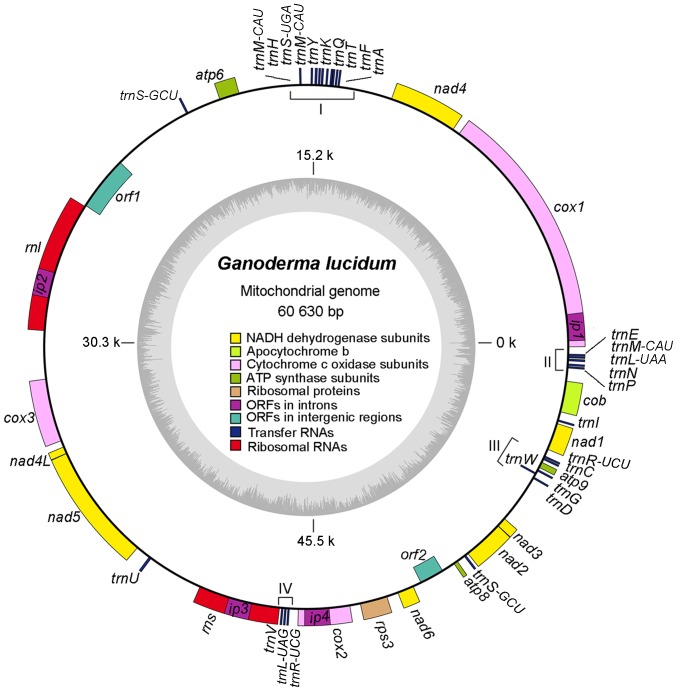
Ideogram of mitochondrial genome of *Ganoderma lucidum*. Genes indicated by closed boxes on the outside of the circle are located on the positive strand, whereas those on the inside (*orf1, orf2*, and *trnW*) of the circle are located on the negative strand. The genome coordinates and GC contents are shown in the inner circle. The genes are colored based on the functional group they belong. The color scheme is shown in the middle of the circle. Four intron proteins, namely, *ip1*, *ip2*, *ip3*, *and ip4*, are drawn within the corresponding genes. Four putative clusters of tRNA genes are indicated with brackets and Roman numerals. For clarity, tRNA genes, which are present as isoforms, are indicated by attaching the anticodon to the gene name.

The mitochondrial genome of *G. lucidum* is a typical circular DNA molecule of 60,630 bp with 26.67% GC content. About 62.69% of the mitochondrial genome of *G. lucidum* contains 50 genes encoding two ribosomal RNAs, 27 transfer RNAs, one ribosomal protein gene *rps3*, 14 genes involved in respiratory chain complexes, four ORFs in the intron of other genes (ip1–4), and two ORFs in the intergenic regions (*orf1* and *orf2*). All of these genes were in the same orientation, except those genes encoding for *trnW*, *orf1*, and *orf2*. Figure 1 shows an ideogram that describes the genome organization and gene classification.

#### Protein-coding gene

According to the annotation results, 15 known protein-coding genes were identified in the mitochondrial genome of *G. lucidum*, including cytochrome c oxidase subunits 1–3 (*cox1*, *cox2*, and *cox3*), apocytochrome b (*cob*), NADH dehydrogenase subunits 1–6 (*nad1*, *nad2*, *nad3*, *nad4, nad4L*, *nad5*, and *nad6*), ATP synthase subunits 6–9 *(atp6*, *atp8*, and *atp9*), and ribosomal protein *rps3* (Table 1). These proteins were highly conserved in sequences compared with those in *T. cingulata*, a wood-degrading basidiomycete. Two ORFs (>100 aa) were found on the negative strand. ORF1 consisted of 677 amino acids, showing similarity to a DNA-dependent RNA polymerase of *M. perniciosa* (Accession No. YP_025889; length = 1028 aa; alignment range, 67–655 aa; identity = 191/656 (29%); e-value = 3e-49). A stop-codon mutation existed at the amino acid position of 569 in ORF1. ORF2 consisted of 391 amino acids and shows similarity to a DNA polymerase II of *P. ostreatus* (Accession No. YP_001504344; length = 720 aa; alignment range, 75–311 aa; identity = 74/327 (23%); e-value = 7e-08).

**Table 1:** Genetic contents in the mitochondrial genome of *Ganoderma lucidum*.

Functional category	Group of genes	Name of genes
Self replication	Ribosomal RNAs	*rns, rnl*
	Ribosomal protein	*rps3*
	Transfer RNAs	*trnA-UGC, trnF-GAA, trnT-UGU, trnQ-UUG, trnK-UUU, trnY-GUA, trnM-CAU* ^a^, *trnS-UGA, trnH-GUG, trnM-CAU* ^b^, *trnS-GCU* ^d^, *trnU-UCA, trnV-UAC, trnL-UAG, trnR-UCG, trnS-GCU* ^e^, *trnG-UCC, trnW-CCA, trnD-GUC, trnC-GCA, trnR-UCU, trnI-GAU, trnP-UGG, trnN-GUU, trnL-UAA, trnM-CAU* ^c^, *trnE-UUC*
Genes for respiratory chain complexes	Cytochrome c oxidase subunits	*cox1, cox2, cox3*
	Apocytochrome b	*cob*
	NADH dehydrogenase subunits	*nad1, nad2, nad3, nad4, nad4L, nad5, nad6*
	ATP synthase subunits	*atp6, atp8, atp9*
ORFs in intronic sequences	Endonuclease with “GIY-YIG” motif	*ip1, ip4*
	Endonuclease with “LAGLIDADG” motif	*ip2, ip3*
ORFs in intergenic regions	DNA-dependent RNA polymerase like	*orf1*
	DNA polymerase like	*orf2*

*trnM-CAU* ^a^, *trnM-CAU* ^b^, and *trnM-CAU* ^c^ represent three *trnM* isoforms with the same anticodon but different sequences. *trnS-GCU^d^* and *trnS-GCU^e^* mean two *trnS* copies with the same sequences.

#### Introns and ORFs in introns

Thirteen introns were found in the protein-coding genes *cox1*, *cox2*, *cox3*, *nad4*, and *nad5*, as well as in *rnl* and *rns* genes. All introns were group I introns, except for the single type II intron *cox1 i6* (Table 2). Group I introns are mobile genetic elements that interrupt protein-coding and structural RNA genes. Group I introns are often invaded by smaller genes that encode proteins, usually called homing endonucleases, with mobility-promoting activities that enable the DNA element to move within and between genomes [27]. Four ORFs (IP1–IP4) that encode such homing endonucleases were identified in the introns of *cox1 i1*, *rnl i1*, *rns i1*, and *cox2 i1*, respectively. IP1 and IP4 were similar in sequence to GIY endonuclease. The conserved GIY-YIG catalytic domain was located in positions 71–157 aa in IP1 with a Pfam e-value of 2.3e-08 and in positions 68–152 aa in IP4 with a Pfam e-value of 9.2e-13. IP2 and IP3 showed identity to homing endonucleases with the conserved motif “LAGLIDADG.” They both had two LAGLIDADG motifs. In IP2, one domain was located in positions 27–130 aa with a Pfam e-value of 7.3e-26 and the other in positions 182–287 aa with Pfam e-value of 2.2e-14. In IP3, one domain was located in positions 44–154 aa with a Pfam e-value of 5.8e-12 and the other in positions 194–290 aa with a Pfam e-value of 1e-06.

**Table 2:** Introns found in mitochondrial genes.

Category for introns	Location of introns	Name of introns
Group I	*cox1*	*cox1 i1, cox1 i2, cox1 i3, cox1 i4, cox1 i5*
	*cox2*	*cox2 i1*
	*cox3*	*cox3 i1*
	*nad4*	*nad4 i1*
	*nad5*	*nad5 i1, nad5 i2*
	*rnl*	*rnl i1*
	*rns*	*rns i1*
Group II	*cox1*	*cox1 i6*

#### Codon bias and tRNA genes

The protein sequences of 15 predicted mitochondrial genes, including *atp6*, *atp8*, *atp9*, *cob*, *cox1*, *cox2*, *cox3*, *nad1*, *nad2*, *nad3*, *nad4*, *nad4L*, *nad5*, *nad6*, and *rps3*, were used to calculate the frequency of codon usage for non-intronic proteins. The codon usage of intronic proteins was calculated using four intronic protein sequences (IP1–IP4). A strong bias of using “A” or “U” at the first and third codon positions was observed (Table 3). Except for the *cob* that starts with the “GUG” initiation codon, all other genes start with the “AUG” initiation codon. Most coding genes end with the preferred UAA stop codon. However, the coding regions of *nad1*, *nad2*, *ip2*, and *ip3* genes end with the “UAG” stop codon. Table 3 shows that the codon usage in *G. lucidum* mitochondrial genes is strongly biased toward codons with “A” or “U” because of the high AU content in the mitochondrial genome. For example, synonymous codons for Leu exhibited a strong usage bias between UUA (13.42% for non-intronic proteins and 7.06% for intronic proteins) and CUC (0% for non-intronic proteins and 0.16% for intronic proteins). The most frequently used codons in non-intronic proteins consisted of Us and As only, including UUA (13.42%), UUU (5.27%), AUA (5.11%), AUU (5.03%), AAU (4.11%), and UAU (3.72%). By contrast, the least frequently used codons, namely, GGC (0.02%), CCC (0.09%), and CCG (0.11%) mainly consisted of Cs and Gs (Table 3). This trend was also similar in intronic proteins. For example, the most frequently used codon in intronic proteins was AAA (10.03%), whereas the least frequently used codons were CCC (0.08%) and CGG (0.08%). Codons such as AAG, CUC, CGC, CGG, CGU, and UGA were not found in the 15 non-intronic proteins, whereas GCG, GUG, CCG, CGC, and UCC were not found in the four intronic proteins. The alternate codon UGA was only used for tryptophan in intronic protein IP4 and was not used as a stop codon for the proteins we analyzed.

**Table 3:** Codon usage and codon-anticodon recognition pattern for tRNA in the mitochondrial genome of *Ganoderma lucidum*.

AA	Codon	% of all codons(non-intronic proteins)	% of all codons(intronic proteins)	tRNA genefound	AA	Codon	% of all codons(non-intronic proteins)	% of all codons(intronic proteins)	tRNA genefound
Ala	GCA	2.47	1.04	+	Pro	CCC	0.09	0.08	−
Ala	GCC	0.52	0.24	−	Pro	CCG	0.11	0	−
Ala	GCG	0.04	0	−	Pro	CCU	1.9	2.25	−
Ala	GCU	3.26	1.20	−	Gln	CAA	2.47	1.85	+
Cys	UGC	0.07	0.16	+	Gln	CAG	0.07	0.40	−
Cys	UGU	0.44	0.80	−	Arg	AGA	1.99	3.53	+
Asp	GAC	0.2	0.32	+	Arg	AGG	0.02	0.16	−
Asp	GAU	2.03	3.69	−	Arg	CGA	0.02	0.08	+
Glu	GAA	2.43	5.78	+	Arg	CGC	0	0	−
Glu	GAG	0.09	0.72	−	Arg	CGG	0	0.08	−
Phe	UUC	3.04	0.32	+	Arg	CGU	0	0.24	−
Phe	UUU	5.27	4.82	−	Ser	AGC	0.04	0.32	++
Gly	GGA	2.69	1.36	+	Ser	AGU	2.88	1.93	−
Gly	GGC	0.02	0.24	−	Ser	UCA	2.97	1.77	+
Gly	GGG	0.68	0.48	−	Ser	UCC	0.11	0	−
Gly	GGU	3.08	3.29	−	Ser	UCG	0.04	0.16	−
His	CAC	0.61	0.64	+	Ser	UCU	2.51	3.13	−
His	CAU	1.31	2.01	−	Thr	ACA	2.6	2.25	+
Ile	AUA	5.11	3.85	−	Thr	ACC	0.22	0.32	−
Ile	AUC	1.4	0.72	+	Thr	ACG	0.04	0.08	−
Ile	AUU	5.03	3.37	−	Thr	ACU	2.69	2.33	−
Lys	AAA	2.93	10.03	+	Val	GUA	3.8	2.09	+
Lys	AAG	0	1.12	−	Val	GUC	0.22	0.24	−
Leu	CUA	0.63	1.52	+	Val	GUG	0.72	0	−
Leu	CUC	0	0.16	−	Val	GUU	1.94	1.93	−
Leu	CUG	0.09	0.16	−	Trp	UGG	1.2	1.20	+
Leu	CUU	0.66	1.36	−	Trp	UGA	0	0.08	−
Leu	UUA	13.4	7.06	+	Tyr	UAC	1.01	0.40	+
Leu	UUG	0.44	0.56	−	Tyr	UAU	3.72	5.54	−
Met	AUG	1.99	1.44	+++	SeC	UGA	0	0	+
Met	GUG	0.02	0	−	*	UAA	0.33	0.16	−
Asn	AAC	0.63	0.80	+	*	UAG	0.04	0.16	−
Asn	AAU	4.11	7.22	−	*	UGA	0	0	−
Pro	CCA	1.57	0.24	+					

Abbreviations: “AA”: amino acid; “+”: one copy of tRNA gene is found; “++”: two copies of tRNA genes are found; “+++”: three copies of tRNA genes are found; “−”: no corresponding tRNA genes are found; “*”: stop codons.

The 27 identified tRNA genes were roughly clustered into four groups (Figure 1). The genes carried codons for all 20 amino acids and a special amino acid, namely, selenocysteine (SeC or U). SeC is the 21^st^ proteinogenic amino acid. SeC exists naturally in all kingdoms of life as a building block of selenoproteins [28]. As shown in Table 3, the numbers of tRNA genes coding for the same amino acid also varied. Three different *trnM-CAU* genes had the same anticodon. Two copies of *trnS-GCU* genes, which exist in a long forward repeat region, and one *trnS-UGA* gene were found for serine. Two different tRNA genes for each leucine (*trnL-UAG* and *trnL-UAA*) and arginine (*trnR-UCU* and *trnR-UCG*) were found. The other 17 tRNA genes had only one copy. For the codons used in the mitochondrion that lack the corresponding tRNAs (indicated with “−”), this result can be attributed to the peculiar wobble mechanisms in fungal mitochondrial genomes, which have one tRNA for each synonymous codon family [29], [30]. All tRNAs have classic cloverleaf structures based on tRNAscan.

#### Repeat regions

The intergenic spacer (IGS) regions reached 37.31% in *G. lucidum* mtDNA and were rich in direct (forward), inverted, and tandem repeats. For the repeat structure analysis, eight forward, two inverted, and three tandem repeats were detected in the mitochondrial genome of *G. lucidum* (Table 4). These repeats were located in the IGS, intron, and coding DNA sequence (CDS) regions, with the IGS regions being the most frequent. Among the eight forward repeats, three (F1, F2, and F3) were particularly long. F1 was the longest repeat with 244 bp and was located in the IGS of *trnS-orf1*, IGS of *trnS-nad2,* and in the partial CDS of *nad2*. Repeat F1 was also conserved in the mitochondrial genome of *T. cingulata.* The presence of repeats is a potential source of recombination. Two direct repeats can lead to a loop-out process giving rise to submolecules, whereas two inverted repeats can lead to a flip-flop mechanism giving rise to inversion (see below). The statues of these repeats need to be analyzed further.

**Table 4:** Distribution of large repeat loci in the mitochondrial genome of *Ganoderma Lucidum*.

No.	Type	Size(bp)		Start (Unit 1)	Location (Unit 1)	Start (Unit 2)	Location (Unit 2)
F1	F^a^	244	19794		IGS^d^ (trnS, orf1)	51844	IGS (trnS, nad2), CDS^e^ (nad2)
F2	F	193	12003		IGS (nad4, trnA)	47271	IGS (cox2, rps3)
F3	F	139	19632		IGS (atp6, orf1)	51681	IGS (atp8, trnS), CDS (trnS)
F4	F	48	30762		IGS (rnl, cox3)	56384	CDS (trnC), IGS (trnC, trnR)
F5	F	40	28536		Intron (rnl)	43579	Intron (rns)
F6	F	34	30726		IGS (rnl, cox3)	56344	CDS (trnC)
F7	F	34	2637		Intron (cox1)	8564	Intron (cox1)
F8	F	31	2677		Intron (cox1)	33564	Intron (cox3)
I1	I^b^	31	13574		IGS (nad4, trnA)	31107	IGS (rnl, cox3)
I2	I	31	27481		Intron (rnl)	56050	CDS (atp9)
T1	T^c^	15×3^f^	47867		IGS (rps3, nad6)		
T2	T	18×2	11223		CDS (nad4)		
T3	T	26×3	15761		IGS (trnM, atp6)		

a F: forward (direct) repeat;

b I: inverted (palindromic) repeat;

c T: tandem repeat;

d IGS: intergenic spacer regions;

e CDS: coding DNA sequence regions.

f For the size of the tandem repeat, ×3 or ×2 means that the same unit repeats for three or two times, respectively. For the IGS and CDS regions, the names of the neighboring genes and the genes are shown in parentheses, respectively. The names of the intron regions are also shown.

#### Sequence similarity to nuclear DNA

**Figure 2:**
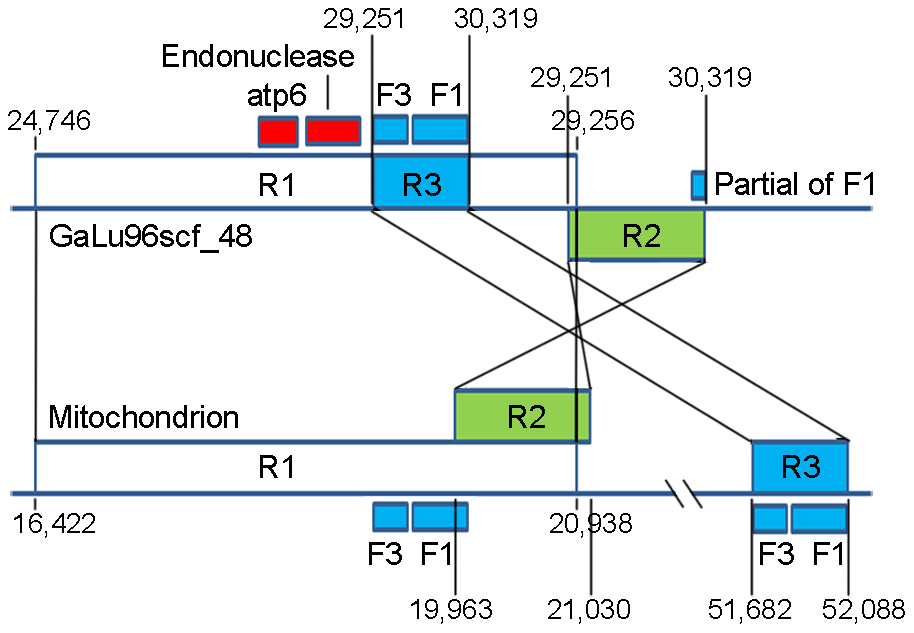
Similarity analysis of sequences between mtDNA and nuclear DNA. The top sequence represents the scaffold 48 from the nuclear genome assembly galu96 and the bottom sequence represents the mitochondrion. The homologous regions can be classified into three regions, namely, R1 (white box), R2 (green box), and R3 (blue box), which are connected with black lines at both ends of the corresponding fragments. The coordinates showing the start and end of each region are also shown. F1 and F3 represent two forward repeats. Endonuclease gene and *atp6* are also indicated (red box) in GaLu96_scf48.

Continuous insertions of mtDNA sequences exist in nuclear chromosomes. To determine if any DNA fragments transfer between the nuclear and mitochondrial genomes, we compared nuclear DNA against mtDNA. The results are shown in Figure 2 and Table S2. As shown, pairs of sequences with a high sequence similarity were found in the nuclear genome (scaffold GaLu96scf_48) and mtDNA, respectively. The sequences consisted of three fragments (R1, R2, and R3) with a total length of 5.5 kb. The percentage of identities for these fragments ranged from 96% to 99%. Fragment R1 (shown in white) was the longest and included two forward repeats, namely, F1 and F3. Fragment R2 (shown in green) included a partial sequence of F1, which is reverse complement to that found in R1. Fragment R3 (shown in blue) consisted of F1 and F3 sequences and was found inside of R1 on both genomes and outside of R1 on the mitochondrial genome only. An extra inverted copy of R2 region was found in the nuclear genome. The origin of these homolog fragments between the nuclear and mitochondrial genome is currently being studied. The comparison of the nuclear DNA fragment with those sequences in GenBank revealed two genes, *atp6*, and a homing endonuclease with a LAGLIDADG motif. The two copies of *atp6* gene in nuclear and mitochondrion were 100% identical.

#### OriC

Ori-Finder (http://tubic.tju.edu.cn/Ori-Finder/) is an online system to find oriCs in bacterial genomes. Three possible oriCs (oriC I, oriC II, and oriC III) were found in the mtDNA of *G. lucidum* by using *Haemophilus*-specific DnaA boxes as references (Table S3). Given the lack of specific DnaA box sequences for fungal mtDNA, the accuracy of the prediction cannot be determined and future experimental validation is needed.

### II. Transcriptome

#### Gene expression and noncoding RNAs in IGS

To determine the expression regulation of mitochondrial genes, we mapped the reads to the genome sequence. The reads, which were 100 bp long, were obtained from the RNA-Seq analysis of the whole transcriptomes from samples collected at three different developmental stages (mycelia, primordia, and fruiting bodies). The RNA-Seq experiments were performed using the library constructed for transcripts with poly(A) tails. Transcripts shorter than 500 bp were removed from the library construction steps. The relative expression levels of the different genes may be biased as a result of the experimental procedures. Nevertheless, for the same genes, their differential expression at the three different developmental stages should be less affected by the experimental procedures adopted, similar to those previously described because the same experimental procedures were applied [31]. The corresponding transcripts of tRNA genes were not included in the libraries subjected to RNA-Seq analysis because their lengths were approximately 80 bp long.

**Figure 3:**
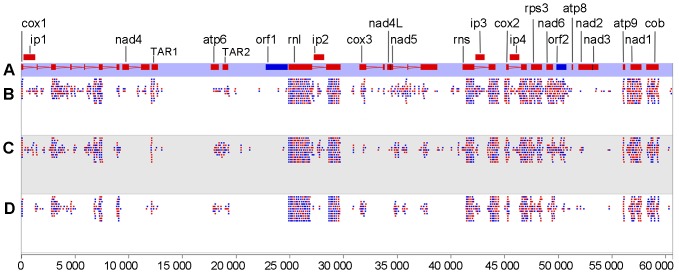
Mitochondrial transcriptome of *Ganoderma lucidum* in three differential developmental stages. Red and blue colors indicate that the genes or reads are on the forward and reverse strands, respectively. (A) Genome organization of *G. lucidum* mitochondria. The intron and exon structures of genes, as well as the names of genes, are shown. Two TAR regions, namely, TAR1 and TAR2, are shown. The mapping of RNA-Seq reads from mycelia (B), primordia (C), and fruiting bodies (D) is also shown. The nucleotide coordinates are shown at the very bottom of the figure.

**Figure 4:**
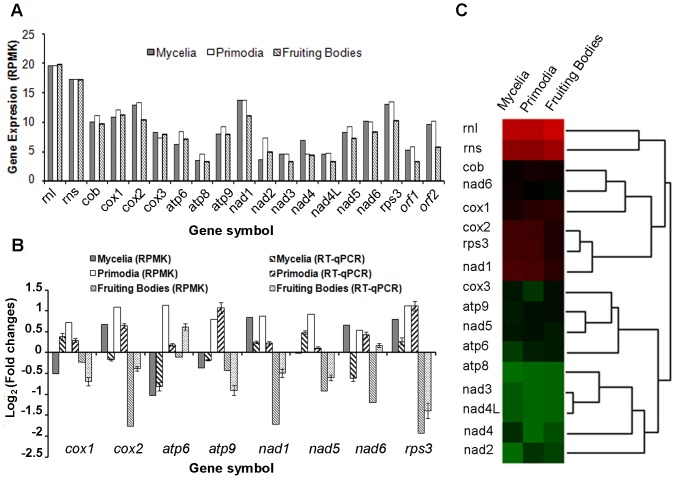
Mitochondrial gene expressions. A. Graphic representation of mRNA abundance for the indicated genes at differential stages analyzed by RNA-Seq. B. Graphic representation of mRNA abundance for the indicated genes at differential stages, as measured by RT-qPCR or RNA-Seq. Error bars denote the standard deviation of the three RT-qPCR replicates. C. Hierarchical clustering of mitochondrial gene expression across three different developmental stages: mycelia, primordia, and fruiting bodies.

A total of 208,286 reads were uniquely mapped to the mitochondrial genome of *G. lucidum*. Figure 3 shows the distribution of the reads along the genome. Four panels are shown, which display the gene structures on the genome (A) and the number of reads from the mycelia (B), primordia (C), and fruiting body (D) stages that are mapped to the genome, respectively. Each dot represents an arbitrary number of reads. The expression levels for all genes were calculated and represented as reads per million per kilobase (RPMK) as previously described [3] (Figure 4A). All of the protein-coding genes were expressed in at least one developmental stage. Except for *cox3*, other protein-coding genes, including *orf1* and *orf2*, had the highest RPMK in either the mycelia or primordial stages, which is consistent with the fact that cellular growth is most active in these two stages. Compared with protein-coding genes, the rRNA genes were expressed at three stages with no difference.

To compare the gene expression levels across the three stages, we normalized the gene expression level in each stage against that of the average of three stages. Eight genes with higher RPMK (*cox1*, *cox2*, *atp6*, *atp9*, *nad1*, *nad5*, *nad6*, and *rps3*) were selected for RT-qPCR analysis to confirm the gene expression levels. As shown in Figure 4B, *cox1*, *cox2*, *atp9*, *nad5*, *and rps3* genes had higher expression levels at the mycelia or primordial stages (> twofold changes). However, *nad1* and *nad6* did not show significantly higher expression levels (< twofold changes). Compared with the results of RNA-Seq, the expression level of *atp6* at the mycelia or primordial stages was lower than that of fruiting bodies.

To determine if functionally related or adjacent genes were co-expressed, we clustered the genes based on their expression profiles across the three developmental stages (Figure 4C). The functionally related genes, such as “*cob, cox1*”, “*atp6*, *atp9*”, and “*nad3, nad4L, nad4*, *nad2*” showed similar expression profiles. Two groups of genes, namely, “*cob, cox1*” and “*cox2, rps3*” were co-expressed and adjacent on the genome. However, its mechanism remains to be elucidated.

Fungal mitochondria were recently reported to produce various RNA molecules derived from IGS regions that do not code for any proteins; thus, they can be considered as noncoding RNA. The expression levels of these noncoding RNAs were similar to those of the protein-coding RNAs [24], [32]. Two TARs, namely, TAR1 and TAR2 were detected to not encode any identified protein or tRNA products. TAR1 was located in the IGS between *nad4* and *trnA-UGC*, whereas TAR2 was between *atp6* and *trnS-GCU* (Figure 3). The expression profile of TAR1 was higher than that of *nad4*, and the expression profile of TAR2 was similar to that of *atp6*. The functions of these TARs remain unknown.

#### Identification of RNA-editing sites

RNA editing is a widespread post-transcriptional molecular phenomenon that can increase proteomic diversity by modifying the sequence of completely or partially nonfunctional primary transcripts. In our results, three editing modifications (one was C-to-U substitution and two were A-to-C substitutions) were identified in the transcriptome of mycelia and primordial stages, but they were not found in the transcriptome of fruiting bodies (Table 5). The editing modification that occurred in RPS3 led to an amino acid change from Leu to Phe; however, that in NAD1 did not lead to an amino acid change. The exact functional consequences of these RNA-editing events remain unknown.

**Table 5:** Identification of RNA-editing sites.

ID	Locus	Gene	Genome	Variants	AAchange	Mycelia	Primordia
Edit 1	44179	rns	A	C	No	509(510)^a^	182(184)
Edit 2	48049	rps3	C	U	Leu to Phe	10(10)	7(8)
Edit 3	56865	nad1	A	C	No	151(151)	78(79)

a The numbers outside the parenthesis represent the numbers of RNA-Seq reads mapped to the position showing the variants, whereas the numbers inside represent the numbers of all RNA-Seq reads mapped to this position.

### III. Comparative Genomic Analysis

#### Molecular phylogenetic analysis

**Figure 5:**
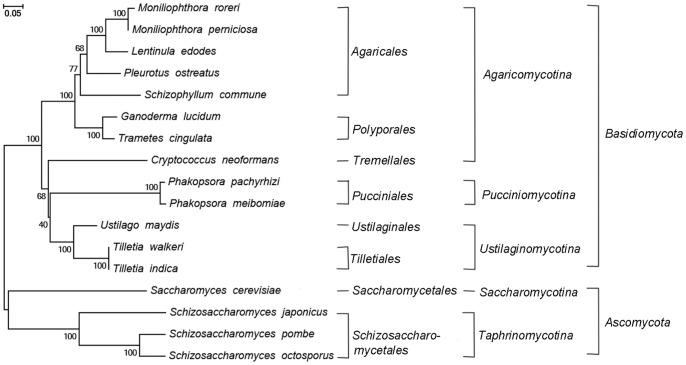
Molecular phylogenetic analysis. Amino acid sequences of the genes *atp6*, *atp8*, *atp9*, *cob*, *cox1*, and *cox2* in *Ganoderma lucidum* and other 16 fungal species were used to construct this tree with the maximum likelihood method implemented in the software MEGA 5.05. The order, sub-kingdom, and kingdom corresponding to each species show their taxonomic classifications. Bootstrap supports were calculated from 1000 replicates.

To understand the evolutionary history of the mitochondrial genome of *G. lucidum*, we identified genes that were present in 16 fungi (including 12 basidiomycetes and four ascomycetes). These genes included *atp6*, *atp8*, *atp9*, *cob*, *cox1*, and *cox2*. The amino acid sequences of these genes were then used for phylogenetic tree construction (Figure 5). The order, sub-kingdom, and kingdom of each fungi species are also shown. *G. lucidum* and *T. cingulata* belong to the order Polypore in the sub-kingdom *Agaricomycotina*, and they are clustered in the same clade associated with posterior probability support of 100%. Figure 5 shows the mitochondrial genomes of the yeast species cluster apart from the mitochondrial genomes obtained from the basidiomycete species. Among the 13 basidiomycete species, *G. lucidum* and *Agaricales* species are phylogenetically closer than other sister clades of *Agaricomycotina*. The phylogenetic relationship is in agreement with the classification of basidiomycetes in fungi.

#### Comparison of genome synteny

**Figure 6:**
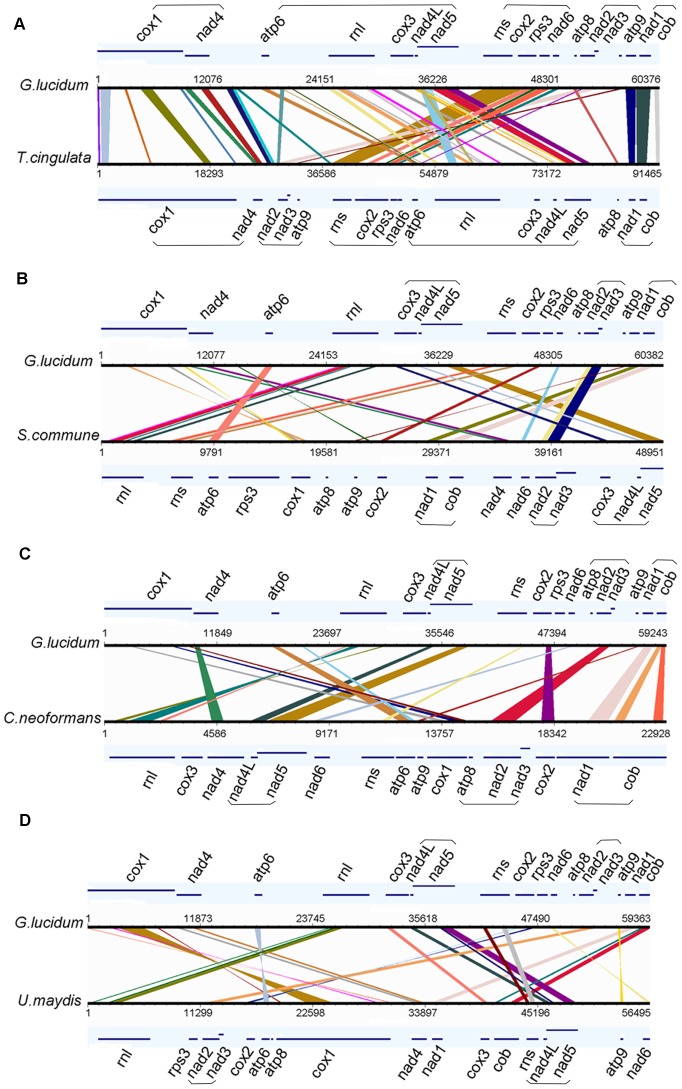
Synteny analysis of mitochondrial genomes. Synteny comparison of genomes between *Ganoderma lucidum* and *Trametes cingulata* (A), *Schizophyllum commune* (B), *Cryptococcus neoformans* (C), and *Ustilago maydis* (D). The genes are represented with lines, and their names are shown next to the corresponding lines. The conserved gene blocks are highlighted with parentheses. The conserved regions are bridged by lines. Genome synteny visualization was performed by GSV, a web-based genome synteny viewer. Co-rearrangement genes are underlined.

Mitochondrial genomes undergo complicated genome rearrangement. To determine if the same phenomenon occurs in the mitochondrial genome of *G. lucidum*, the mitochondrial genomes of *T. cingulata*, *S. commune*, *C. neoformans*, *and U. maydis* were selected for genome synteny analysis with that of *G. lucidum*. These four genomes were selected as representative species of *Polyporales*, *Agaricales*, *Tremellales*, and *Ustilaginales*, respectively. As shown in Figure 6, the sequences of protein-coding genes were clearly highly conserved, but the relative position of the genes underwent rather complex rearrangement among the genomes. The result did not show significant overall synteny of the protein-coding genes, even between species in the same order (such as *G. lucidum* and *T. cingulata*). For *G. lucidum* and *T. cingulata* (Figure 6A), their mitochondrial genomes could be divided mainly into four blocks (block 1: *cox1-nad4*; block 2: *atp6-rnl-cox3-nad4L-nad5*; block 3: *rns-cox2-rps3-nad6*; and block 4: *nad1-cob*). Blocks 1 and 4 showed no rearrangement, whereas blocks 2 and 3 showed order change, which resulted in an inversion. Another minor rearrangement occurred in the *atp8* and *nad2-nad3-atp9* groups. In *G. lucidum*, the *nad2-nad3-atp9* group was located between *atp8* and block 4, whereas in *T. cingulata*, the location was between blocks 1 and 3. Specific genes stayed together with no rearrangement. For example, the gene pairs (*nad2–nad3* and *nad4L–nad5*) were preserved in all of the five mitochondrial genomes analyzed (Figures 6A, B, C, and D). Similarly, no rearrangements were observed between genes *nad1-cob* in the four *Agaricomycotina* species (Figures 6A, B, and C). This finding suggests that the presence of a purifying force keeps these genes physically clustered together in the genome.

## Discussion

Mitochondrial genomes are widely used in evolutionary and population studies. Given that *G. lucidum* is a complex species and no effective methods are available to differentiate the sub-species, mitochondrial genome sequences may prove useful for species determination. Mitochondria have an important function in supporting cellular growth. Identification of the gene contents and elucidation of their expression regulation will provide clues for better production of active compounds. This study extends our recent work on whole genome sequencing and transcriptome analysis of *G. lucidum* strain CGMCC5.0026 [3] and provides a detailed analysis of the mitochondrial genome of *G. lucidum*.

The mitochondrial genome of *G. lucidum* is significantly more compact than that of *T. cingulata*, which may be attributed to the loss of intronic sequences. The mitochondrial genome size is moderate compared with that of the published mitochondrial genomes of basidiomycetes, which range from 24,874 bp (*Neurospora crassa*) to 121,394 bp (*L. edodes*). The difference in genomic size is caused by the variation in intergenic regions and the presence of hypothetical proteins [11]. For example, although both intron and intronic ORFs can be found in the mtDNA of *G. lucidum*, *P. ostreatus*, and *T. cingulata,* the number of introns differ. A total of six, nine, and 15 introns were identified for *cox1* gene in *G. lucidum*, *P. ostreatus*, and *T. cingulata,* respectively. By contrast, only one intron was identified for rRNA genes in *rnl* and *rns* of *G. lucidum* mtDNA. This result was also supported by the transcriptome data (Figure 3). In *T. cingulata* mtDNA, *rnl* was split apart by six introns; therefore, more intergenic and intronic regions contributed to the size of *T. cingulata* mtDNA (91,500 bp) [16]. The size was larger than that of *P. ostreatus* (73,242 bp) [33] and *G. lucidum* (60,630 bp).

The mitochondrial genome of *G. lucidum* contains genes that are commonly found in other genomes. However, its genomes contain a few unique features. First, one DNA-dependent RNA polymerase (*rpo*) and one DNA polymerase (*dpo*) candidate genes, namely, *orf1* and *orf2*, respectively, were identified. These RNA and DNA polymerases are also found in reported fungal mitochondrial plasmids and mtDNAs [33], [34], [35], [36]. Both *orf1* and *orf2* were located on the negative strand. A stop codon mutation was detected at the amino acid position of 569 in ORF1, which may be a pseudogene. Second, a special tRNA gene encoding SeC was identified in *G. lucidum* mtDNA. SeC is present in several enzymes (for example, glutathione peroxidases, thioredoxin reductases, glycine reductases, etc.). As of this writing, the *trnU* gene is rarely identified in other known basidiomycete mtDNAs. This phenomenon may reflect one of the distinct biological processes in *G. lucidum*, which is known as a medicinal mushroom. We also observed the presence of two fragments showing high degree of sequence similarities in the nuclear genome and the mitochondrial genome, respectively. The nuclear genome had an extra inverted copy of the R2 region with a partial F1 repeat sequence. These regions contained a putative endonuclease. We may hypothesize that the forward repeat and the endonuclease might be derived from an ancient transposon, which excises the fragment from the nuclear genome and forms a sub-molecule that is inserted into the nuclear genome. A comprehensive analysis of similar sequences between fungal nuclear and mitochondrial genomes is needed to test this hypothesis.

In transcriptome analysis, two potential drawbacks exist in this set of data. First, the library preparation was toward RNA with a length >500 bp and having a poly(A) tail. Thus, transcripts that did not have poly(A) tails were likely to be excluded from the library. In addition, some reads were generated from the nuclear genomes that were subsequently mapped in the mitochondrial genome. However, transcripts that were believed to be excluded from the library were frequently found. This finding is likely due to the internal poly(A) sequences in the transcripts and the nonspecific selection of transcripts without poly(A) tails. Given that the RNA-Seq reads are generated using a sequencing library constructed via a non-strand-specific library construction method, we cannot determine the strand-specificity of the reads. The relative expression levels among different genes are more likely to be biased. Thus, we did not draw any conclusion regarding the expression levels among different genes. However, the expression levels for the same genes at the three different developmental stages were comparable because the transcripts derived from the same genes were treated in the experimental conditions. The relative expression levels were validated using RT-PCR experiments. The expression profiles for the same genes across different stages from the two different platforms were similar. All other sequences in the mitochondrial genome were compared with the nuclear genome to eliminate possible nonspecific mapping.

Based on the transcriptome data, several interesting features were observed. First, the mRNA abundances of most genes were higher in the mycelia or primordial stages. This observation was consistent with stages having high energy needs for rapid cellular growth and development. Second, “*cob, cox1*” and “c*ox2, rps3*” were found to be co-expressed and adjacent on the genome. The functional significance of these observations needs to be studied further. Third, we identified two potential long noncoding (lnc) RNAs. Recent studies showed that lncRNA can regulate gene expression through a wide variety of mechanisms [37]. Ascertaining if the expressions of particular mitochondrial genes are regulated by lncRNA would be highly interesting.

Fungal mitochondrial genomes do not show an overall high degree of synteny [15]. At the class level, some fungal mitochondrial genomes from ascomycetes show significant synteny [38]. In basidiomycetes, the number of sequenced mitochondrial genomes was significantly limited and the analysis of the content and order of genes did not show overall synteny. The protein-coding genes were highly conserved between the mitochondrial genomes of *G. lucidum* and *T. cingulata*, but the order of some genes was rearranged. The rearrangement that produces duplications, inversions, and deletions in noncoding regions can mediate genome evolution. Thus, the different sequences of introns or IGS will be developed as DNA markers for species identification. Some regions such as *nad2–nad3* and *nad4L–nad5* were resistant to rearrangement. The mechanisms supporting their linkage remain to be studied.

This study provides valuable information on the mitochondrial genome composition and the differential expression of mitochondrial genes across three developmental stages. This research provides possible genetic markers for the differentiation of closely related *G. lucidum* sub-species or strains. Our findings lay the foundation for a complete understanding of the functions of *G. lucidum* mitochondria in the growth and development of *G. lucidum* cells.

## Supporting Information

Table S1 **Primer sequences for PCR or RT-qPCR.**
(XLSX)

Table S2 **Similarity analysis between sequences from mtDNA and nuclear DNA.**
(XLSX)

Table S3 **Prediction of oriC region.**
(XLSX)
